# Pediatric Palliative Care for Children with Cancer in a Children's Tertiary Hospital in China: Six-Year Experience of a Pediatric Palliative Care Service

**DOI:** 10.1089/pmr.2020.0030

**Published:** 2021-01-06

**Authors:** Anan Zhang, Ling Bing, Qiang Mi, Fen Zhou, Jianmin Wang

**Affiliations:** Department of Hematology/Oncology, Shanghai Children's Medical Center, Affiliated to Shanghai Jiaotong University School of Medicine, Shanghai, China.

**Keywords:** adolescent, child, China, infant, neoplasms, palliative care

## Abstract

***Background:*** Pediatric palliative care (PPC) does not meet current needs, particularly in low- and middle-income countries.

***Objective:*** We evaluated the first PPC team to serve patients with cancer in a tertiary children's hospital in China.

***Design:*** Single-center retrospective study.

***Setting/Participants:*** The core team members included oncologists, nurses, and a social worker. The team delivered palliative care through the outpatient clinic, consultations, a 24/7 hotline, and a hospice room located in the observation ward. Patients were referred by pediatric oncologists. We analyzed data for 92 children (54 boys and 38 girls; aged 7 months to 16 years) who required palliative care from August 2012 to August 2018. The most common primary diseases were leukemia and neuroblastoma.

***Measurements:*** We investigated the time from referral to death, symptoms during the prior month, the effects of informing children above eight years, and family satisfaction.

***Results:*** Among 88 deaths, the median time from referral to death was 17 (range 1–218) days. Most children had multiple symptoms (mean ± standard deviation 4.2 ± 3.2 per child). The most common symptoms in the last month of life were pain, loss of appetite, fatigue, fever, and dyspnea. Children above eight years who were not informed about their condition experienced more anxiety or depression. All families were satisfied with the services.

***Conclusions:*** The palliative care counseling team is feasible and could be complementary to conventional medicine in caring for children with life-limiting illnesses. This model has an important role in PPC in China or developing countries with scarce medical resources.

## Introduction

**FIG. 1.**
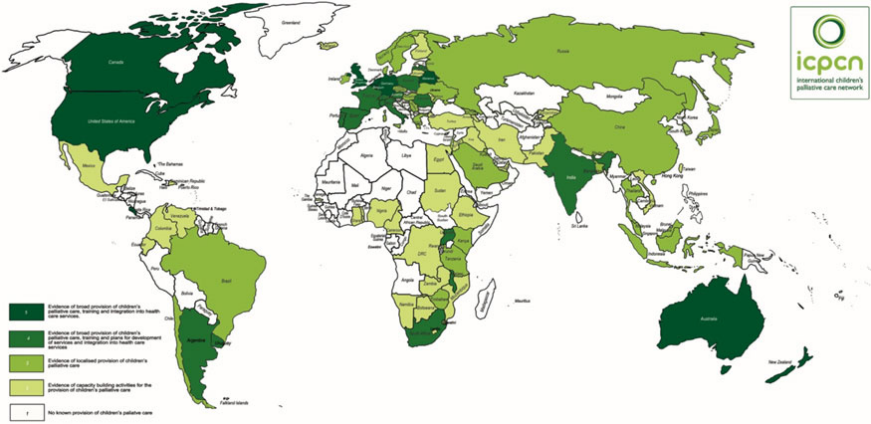
Map showing ICPCN's estimated levels of children's palliative care worldwide. (From ICPCN (International Children's Palliative Care Network, http://www.icpcn.org/).

Pediatric palliative care (PPC) is defined by the World Health Organization (WHO)^1^ as “the active total care of a young person's body, mind, spirit, and family, during a life-limiting diagnosis.” The United Nations has pointed out that palliative care is a basic human right for children, and the World Health Assembly^2,3^ resolved that “palliative care is a core component of universal health coverage and a key element of quality health care.” However, PPC and pain relief endeavors are far from meeting current needs. As many as 21 million children globally require PPC each year, including 8 million who could have problems that require specialist PPC.^4^ Moreover, 80% of the global need is in low- and middle-income countries (LMICs), but the majority of PPC is provided in high-income countries (Fig. 1).^5,6^

PPC models include independent hospices, hospital palliative care wards, palliative care counseling teams (PCCTs), and community and home-based palliative care services, among others. Successful service models in developed countries are not fully applicable to developing countries. The barriers and challenges associated with palliative care development for PPC in LMICs include a lack of medical staff, lack of specialist education, lack of policies, lack of integration into the health system, and a lack of awareness and available services.^7,8^ Therefore, a feasible PPC service model in the context of limited resources in LMICs is crucially needed. Our children's tertiary hospital is one of the largest pediatric hematology/oncology centers in China. We have a considerable need for palliative care, which urged us to start a PPC service. This study investigated the effectiveness and feasibility of our PCCT, summarizing our six years of experience.

## Methods

The manuscript was approved by IRB (SCMCIRB-W2019007).

### Composition of the PCCT

Our hematology/oncology department started receiving a monthly grant of $500 from St. Jude Children's Research Hospital in 2008, specifically to pay a physician's salary to provide part-time PPC services. Initially, this physician taught herself the WHO document “Cancer Pain Relief and Palliative Care in Children-1998” and started providing cancer pain management in our department. In 2010, with funding from Project HOPE (Health Opportunity for People Everywhere), two senior nurses in our department went to National Cheng Kung University Hospital in Taiwan to study palliative care. In 2012, the addition of a social worker contributed to the eventual formation of our PCCT.

Our core PCCT members now comprise two attending physicians, two fellows with experience in pediatric oncology, two senior and two junior nurses in hematology/oncology, and one medical social worker. All work part time and obtained a qualified certificate of palliative care job training issued by Shanghai Municipal Health Commission, which is an adult palliative care continuing education program in Shanghai. Two physicians obtained a certificate of Education in Palliative and End-of-Life Care-Pediatrics training, and two nurses obtained a certificate of End-of-Life Nursing Education Consortium-Pediatric training.

The PCCT performed symptom assessment and management, communication, treatment goal identification, care plan development and implementation, and psychosocial support for children and caregivers. To improve the professionalism of new members, the PCCT studied the “WHO Guidelines on the Pharmacological Treatment of Persisting Pain in Children with Medical Illnesses,” “A Really Practical Handbook of Children's Palliative Care,” and “Oxford Textbook of Palliative Care for Children” and regularly discuss cases regarding the quality of the PPC.

The team also provided training and support for clinical frontline medical staff in the department, going through the Good Pain Management training every year, a mandatory course for new employees in our hospital. Every two years, a two-day PPC workshop was held in our department, funded by Project HOPE. All new medical staff in our department participated, learning the PPC concepts and practices through lectures, case discussions, and role-playing. We invited experts from Taiwan, Hong Kong, St. Jude Children's Research Hospital, and MD Anderson Cancer Center, among others. This year, with funding from Project HOPE, we completed a 20-episode medium-level online PPC course open to all staff at our hospital, which was designed for bedside practice of PPC by medical staff with work experience.

Because of a manpower shortage, most PPC in our department was provided by the general medical staff, including pain and symptom assessment and management, patient education, and communication. Volunteers provided convenient services and various activities for children. The PCCT only focused on complex challenging cases and provided consultations for children without malignancies who required PPC in other hospital departments. This joint care model provided experience for the PCCT, and the children received PPC in the original department.

### Patients

This study retrospectively analyzed children who received PCCT services in our hospital from August 2012 to August 2018. We retrieved data from the hospital clinical data system. Symptoms were based on reports by the patient or their parents. The last follow-up was in December 2018.

### Specific services

The physician was responsible for judging and communicating the patient's condition, discussing treatment goals with the patient/family, managing symptoms, and coordinating care plans. Nurses provided regular follow-up and nursing guidance. Physicians and nurses provided general emotional and social support; only one social worker served the entire hospital and could only offer professional psychosocial support and interventions.

Children were referred by oncologists for refractory symptoms, psychosocial needs, or end-of-life care. Referrals for end-of-life care required approval by two experienced chief physicians to confirm that the child's condition was difficult to cure. When the end-of-life care referral was accepted, the parents signed an informed consent form to ensure that they understood that the goal of PPC is to improve quality of life. The family also filled out a basic information form, which included the family decision maker(s), caregiver(s), major concerns, and the medical care expected by the patient/parents/caregivers. We also explored the parents' perceptions of the condition. We asked the family how they would like to discuss the condition with the child and, for children above eight years, how well the child understood the disease.

The PCCT provided services through a PPC clinic once per week, ward consultations, 24/7 telephone support, and a hospice room called “Blue Planet,” an independent and quiet family-style room located in our observation ward that allows family members and friends to visit. The team assisted the family in preparing all the necessary items before the child died. Parents completed a satisfaction questionnaire comprising four numeric and three open-ended questions 1 month after the child died (Appendix Table A1).

## Results

### General data

The PCCT received a total of 103 children from August 2012 to August 2018. We excluded four children who were referred for refractory pain and seven referred for end-of-life care due to incomplete records. Thus, 92 patients (54 boys and 38 girls) were enrolled in this study. The median age at referral was 71 months (range 7 months–16 years). The most common primary diseases were neuroblastoma (*n* = 27, 29.0%), acute lymphoblastic leukemia (*n* = 21, 22.6%), and acute myeloid leukemia (*n* = 15, 16.1%). Other diseases included brain tumors, rhabdomyosarcoma, myelodysplastic syndrome, lymphoma, osteosarcoma, primitive neuroectodermal tumors, and other malignancies.

### Time to death and reasons for referral

Of the 92 children, 4 were alive in December 2018. Among the 88 deaths, the median time from referral to death was 17 days (range 1–218 days).

Reasons for referral included newly diagnosed malignancy (*n* = 5; 5.4%), tumor relapse or refractory tumors (*n* = 76; 82.6%), and serious complications, such as intracranial hemorrhage or respiratory failure (*n* = 11; 12%).

The place of death included home (*n* = 28; 31.8%), the hospice ward (*n* = 32; 36.4%), the local hospital (*n* = 20; 22.7%), the oncology ward (*n* = 5; 5.7%), and the emergency department (*n* = 3; 3.4%).

### Symptoms and interventions

**FIG. 2.**
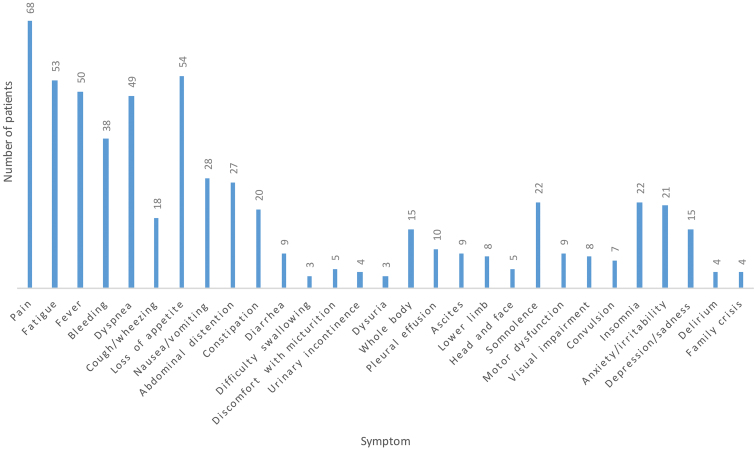
Incidence of the main symptoms in 88 children before death.

The symptoms of children 1 month before death are shown in Figure 2. Most children had multiple symptoms (mean ± standard deviation [SD] 4.2 ± 3.2 per child). The most common symptoms were pain (median pain score 7, range 2–10 points), loss of appetite, fatigue, fever, dyspnea, edema/effusion, and bleeding (Fig. 2). The most commonly used medications and nonpharmacological interventions are given in Table 1. Owing to refractory symptoms, 12 patients used sedation. Based on the physician and parents' preferences, all of these patients received intermittent and moderate sedation (i.e., intermittent use of short-acting agents necessary to achieve symptom relief).

**Table 1.** The Most Commonly Used Medications and Nonpharmacological Interventions

Interventions	No. of patients	Proportion (N = 88), %
Pharmacological		
NSAIDs	86	97.7
Opioid analgesics	60^a^	68.2
Laxatives	28	31.8
Dexamethasone	22	25
Benzodiazepines/phenobarbital/hydrated chloral	18	20.5
Diuretics	16	18.2
Antibiotics	15	17
Nonpharmacological		
Oxygen inhalation	22	25
Nebulization	13	14.8
Palliative radiotherapy	6	6.8
Gastric tube	3	3.4
Catheter	2	2.3
Ventricular drainage	1	1.1

^a^ Fifty-seven patients used morphine.

NSAIDs, nonsteroidal anti-inflammatory drugs.

Only two patients with anxiety/depression took psychiatric medicines. Others with emotional distress were treated with supportive interventions, including listening, relaxation techniques, expressive therapy, and family education.

### Breaking bad news to children

Among the 30 children above eight years, 2 were completely aware of their condition at the time of referral; 16 had families (54.8%) that were unwilling to tell the truth to their children. One child asked us to tell his parents about his condition for him. Eventually, 16 children passed away without understanding their condition. The remaining 12 families were not sure, but finally chose to talk to their children frankly. Ten families asked the physician to break the bad news to the child. Upon learning about their condition, one boy was very depressed until he passed away and one girl became more anxious, often felt overwhelmed, and implored the physician for aggressive treatment. Among the 14 families with a child who had learned of the disease, 6 had religious beliefs (*n* = 2 Buddhism, *n* = 4 Christianity). The children who were not aware of their condition were more frequently anxious or depressed (41.2%, 7/17) compared with those who were aware of their condition (14.3%, 2/14). All families with religious beliefs chose to have an open conversation with the children about their condition and thought the child died peacefully.

### Satisfaction questionnaire

We started using the satisfaction questionnaire as feedback in 2016 (Appendix Table A1). Of the 40 families given the questionnaire, 38 responded for a 95% response rate. All families scored a 4, which meant they were satisfied with the PCCT service.

## Discussion

Access to palliative care is scarce for children in LMICs, and it is even more scarce in mainland China.^5,9^ Our institution was the first to provide PPC in a hospital in mainland China.

The European Society for Medical Oncology has reported that the mean (range) time from referral to death is 21 (14–45) days for inpatients and 90 (40–150) days for outpatients in adult PPC.^8^ For our patients the time frame is 17 (1–218) days. However, the ideal referral time is 6–24 months before death.^10^ Many studies have confirmed that the timely integration of palliative care into routine oncological care has many advantages, including improvements in physical and psychological symptoms, quality of life, and prognosis, as well as reducing costs.^11–14^ However, there are many reasons for delaying a referral. First, the parents and medical staff are reluctant to accept the term “palliative care” because it implies that they are “giving up.”^15^ Second, it is sometimes difficult to estimate the clinical prognosis. Third, family members are slow to understand the disease prognosis compared with the medical staff.^16–18^ As in many countries, “death” is taboo in China and clinicians tend to deliver overly optimistic prognoses. In contrast, in traditional Chinese culture, Confucianism, Taoism, and Buddhism all lead people to accept death. A previous study on Chinese preferences for achieving a good death showed that preparations for death are important.^19^ In clinical work, it is not uncommon for Chinese parents to request the truth from physicians so that they can make appropriate arrangements. For these reasons, we would recommend medical staff be more open to discussing the prognosis with families and improve their communication skills with empathy.

In our hospital, a staff shortage was also a prominent reason for late referrals. Owing to limited manpower, it was difficult to involve the PCCT at the time of diagnosis for every patient. However, to maximize benefits, the PCCT trained all first-line medical staff in the appropriate skills. We also defined referral indications, including refractory symptoms, psychosocial needs, and end-of-life care, which could initiate the PCCT and facilitate a balance between patient needs and medical resources. We previously showed that training the first-line medical staff does not increase their burden, but increased their confidence in clinical work.^20^ Therefore, by providing first-line medical staff education and appropriate indicators, a small PCCT should still be capable of meeting the needs of most patients among scarce resources.

Children with cancer experience severe symptoms at the end of life.^21^ The mean (±SD) number of symptoms per patient in the last week of life has been estimated as 11.1 ± 5.6.^18^ The fewer number of symptoms per patient in this study could be explained by some patients having left Shanghai to return to their hometowns and reporting very few symptoms, potentially due to the inconvenience of contacting us or the treatments provided by local hospitals. In addition, we did not perform regular follow-ups and relied completely on patient reports. It is highly likely that we only counted symptoms that the most distressed patient/parents reported or that were considered treatable by the medical staff. In 2017, a nurse in our institution began assessing patient symptoms every week, which led to an increase in the number of symptoms reported.

At first, we assessed symptoms using the Memorial Symptom Assessment Scale 10–18Y and the Chinese version of the Pediatric Patient-Reported Outcomes Measurement Information System.^22,23^ However, these tools contained too many items to be completed by exhausted patients/parents and busy medical staff and we stopped using them. Therefore, a shorter but sensitive symptom screening tool is urgently needed.

Psychological symptoms are very common in children with cancer and children facing imminent death. Among children with cancer, 36% are reported to have psychological or emotional symptoms, and 22% have both physical and psychological symptoms.^24^ In our study, 44% (39/88) of patients reported psychological symptoms. In addition, four patients reported a family crisis, such as parental divorce. However, our assessment of psychological symptoms was not standardized. A psychiatrist diagnosed depression/anxiety in only two children; other assessments relied on obvious behavioral changes or self-assessments. Distinguishing between a normal concern or sadness about the disease and a pathological mental illness was difficult, even when a social worker was included in the PCCT. Furthermore, it was more difficult to assess psychological symptoms in telephone follow-ups than physical symptom assessments. Therefore, our estimation of psychological symptoms may be inaccurate. Another challenge was the lack of mental and psychiatric professionals, which resulted in a very low referral rate to psychiatrists.

PPC follows all of the principles of adult palliative care, which includes the right to be informed about their condition and the imminence of death.^2^ Children want parents to be honest and frank about the disease and to include them in decisions.^25^ Pretending not to know about the condition only aggravates the child's anxiety, loneliness, and distrust.^26–28^ Kirk and Pritchard compared health-related decisions made by 9-, 14-, 18-, and 21-year-old children with illnesses and found that even a 9-year-old child could make meaningful decisions.^29^ Based on that study, we selected patients aged above eight years to investigate whether parents were willing to inform the children of their illness. Regrettably, most parents avoided talking about this issue. At the end of life, parents' attitudes toward death could hinder the child's wishes.^30^ In contrast, after the child's death, the family often regretted not telling the child the truth.^31^ Upon learning about their illness, most children at our institution faced the situation calmly. Two children experienced emotional distress after learning about the illness; they had both been transferred to the hospice ward without knowing about their parents' decision to stop curative treatment. When they finally learned of their condition, they felt abandoned. This reminded us to break bad news to the child earlier and involve the children in treatment decisions if appropriate. In addition, although we do not know why, we found that religious belief had a positive effect on children and families in the face of death. In the future, parents, children, and medical workers should endeavor to establish more open communication and seek psychological and spiritual support.

Although adults often die in nursing institutions, children often die in tertiary hospitals where they were diagnosed. In China, hospice care is managed and promoted by the community health association. Siouta et al. pointed out that the best palliative care service should include multiple modes of service, from hospitalization to home care.^32^ In our experience, tertiary hospitals are an important part of the palliative care service system. However, the shortage of medical resources in tertiary hospitals is a problem. The PCCT in our hospital represented a preliminary attempt to establish dedicated PPC. Although many aspects of our PPC lacked rigor, the families were satisfied. In addition, it did not require much additional investment other than hardware because our PCCT runs like a volunteer team; the members do not get paid by the hospital for the palliative care work. After training, with support from the PCCT, the frontline medical staff in our department could meet most of the patients' needs. Thus, the workload of the PCCT was not great and guaranteed feasibility. Thus, we infer that a PCCT is an economical means to starting PPC in areas with limited resources, such as China.

A limitation of this study is that it is a single descriptive study of PPC in the hematology/oncology department at a children's tertiary hospital in Shanghai and may not be generalizable to all PPC settings. Another limitation is that little information was available about parents, caregivers, or siblings. In addition, because this retrospective study relied on medical records, we could not determine whether the symptoms were reported by the patients or parents. Finally, we lacked information from the PCCT on the quality of PPC provided by the first-line medical staff.

Our experience indicates that the part-time PCCT is feasible and able to provide the palliative care services and end-of-life care for children with life-limiting illnesses. Overall, the families were satisfied with this care. This model could play an important role in PPC services in China and other developing countries with insufficient medical resources.

## Abbreviations Used

LMICs: low- and middle-income countries
NSAIDs: nonsteroidal anti-inflammatory drugs
PCCTs: palliative care counseling teams
PPC: pediatric palliative care
SD: standard deviation
WHO: World Health Organization

## Appendix

**Appendix Table A1.** Satisfaction Questionnaire

To continuously improve the quality of our palliative care service, we invite you to provide us with valuable opinions and suggestions. Please take a few minutes to give us some feedback, and thank you for your cooperation.			
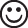
*For the following questions, please check the box that matches your situation. Score 1 means “satisfactory,” 0 means “just ok,” and -1 means “not satisfactory.”*			
	−1	0	1
Are you satisfied with the measures we've provided to relieve symptoms?			
Are you satisfied with the guidance we provided about how to take care of your child?			
Are you satisfied with the psychological support we provided to your children?			
Are you satisfied with the support we provided for your family?			
*Here are some open-ended questions where you can express anything according to your situation.*			
In the final stages of your child's life, what was your biggest concern?			
Which work of the Palliative Care Team did you find most helpful?			
Any suggestions for our Palliative Care Team?			
